# Optimising child and adolescent mental health care – a scoping review of international best-practice strategies and service models

**DOI:** 10.1186/s13034-023-00683-y

**Published:** 2023-12-07

**Authors:** Reinhard Jeindl, Viktoria Hofer, Christian Bachmann, Ingrid Zechmeister-Koss

**Affiliations:** 1 HTA Austria – Austrian Institute for Health Technology Assessment GmbH, Vienna, Austria; 2 Department of Child and Adolescent Psychiatry, University of Ulm, Ulm, Germany; 3 Wiener Gesundheitsverbund, Vienna, Austria

**Keywords:** Child and adolescent mental health, Child health services, Adolescent health services, National strategies, Prevention and care models, Child and adolescent psychiatry, Mental health services, Child and adolescent mental health services, School mental health services

## Abstract

**Background:**

Psychiatric disorders are among the most common health problems in children and adolescents, with a recent prevalence rise due to the COVID-19 pandemic. The increasing demand for service provision in this patient population, together with infrastructural, financial and staff limitations in child and adolescent mental health services, calls for an adaptation/advancement of current models of service provision. This review offers an internationally informed overview of best-practice child and adolescent mental health (CAMH) strategies and care models, with the aim of assisting decision-makers in implementing topical CAMH care models.

**Methods:**

Using a pre-defined structured search strategy, we aimed to identify core topics within published CAMH strategies and care model documents from seven countries within the Global North, which represented a range of differing healthcare systems, geographical regions, and public health traditions. From the retrieved documents, we then systematically extracted data in an iterative process, and summarised these narratively by applying qualitative content analyses.

**Results:**

Our search retrieved the following key components of CAMH strategies: awareness-raising activities, prevention/promotion, detection, treatment, telemedicine, care pathways, transitional psychiatry, vulnerable patient groups, user participation, infrastructure, workforce development, implementation, digital case management tools, and data acquisition/research. Recommendations for CAMH care organisation often followed a public mental health approach, with a focus on mental health promotion, cross-sectional organisation, and funding of CAMH care services. As key principles of best-practice CAMH care models, we identified increased flexibility of care settings, early intervention, and a strengths-oriented approach, with overarching mental health services research alongside.

**Conclusion:**

In order to design robust models of CAMH care and to mitigate current shortcomings, actions on the policy level (e.g., CAMH strategy development with a focus on mental health promotion, installation of cross-sectoral governance), at the organisational level (e.g., re-organisation of treatment settings and pathways of care) and at the individual level (e.g., user involvement, workforce development) are recommended. To this purpose, we strongly advocate the use of cross-sectoral and participatory approaches for CAMH care structures with accompanying health services research.

## Central message

Cross-sectoral, participatory approaches, which focus on the educational sector as well as on prevention and workforce development strategies, have a great potential to improve the mental health of children and adolescents.

## Background

Mental disorders are one of the most common conditions among children and adolescents worldwide, with a prevalence between 9 and 22% [1]. The mental health of children and adolescents was further affected substantially by the COVID-19 pandemic, with a significant reduction in health-related quality of life and a greater extent of depressive symptoms, anxiety and stress reactions [2]. The increasing need for care in this group of patients, with concurrent staffing and infrastructural limitations in child and adolescent psychiatric care, leads to considerations to adapt current models of care provision and delivery.

In this study, we aim to provide an overview of international child and adolescent mental health (CAMH) care strategies and models (e.g., identifying elements of care, coordination, professional groups involved, comprehensive mental health strategy). Mental health care may include prevention, treatment, rehabilitation, diagnostic services, and other types of support. Effectiveness analyses of individual care components, or an assessment to what extent recommendations are already implemented in different countries, are not within this paper’s scope. Our results should support decision-making in the further development of CAMH care structures.

## Methods

### Literature search of national strategies

Between April and June 2022, we conducted a structured hand search for national strategies and models addressing CAMH care and prevention in the following online resources and databases: websites of national ministries of health, websites of national public health institutions, Google (Scholar), World Health Organization MiNDbank [3], Europe encyclopedia of National Youth Policies YouthWiki [4]. For the literature search, various keywords relating to CAMH were combined with the respective country as well as with relevant keywords such as strategy, model, care pathway, prevention.

### Selection of countries and national strategies

**Fig. 1:**
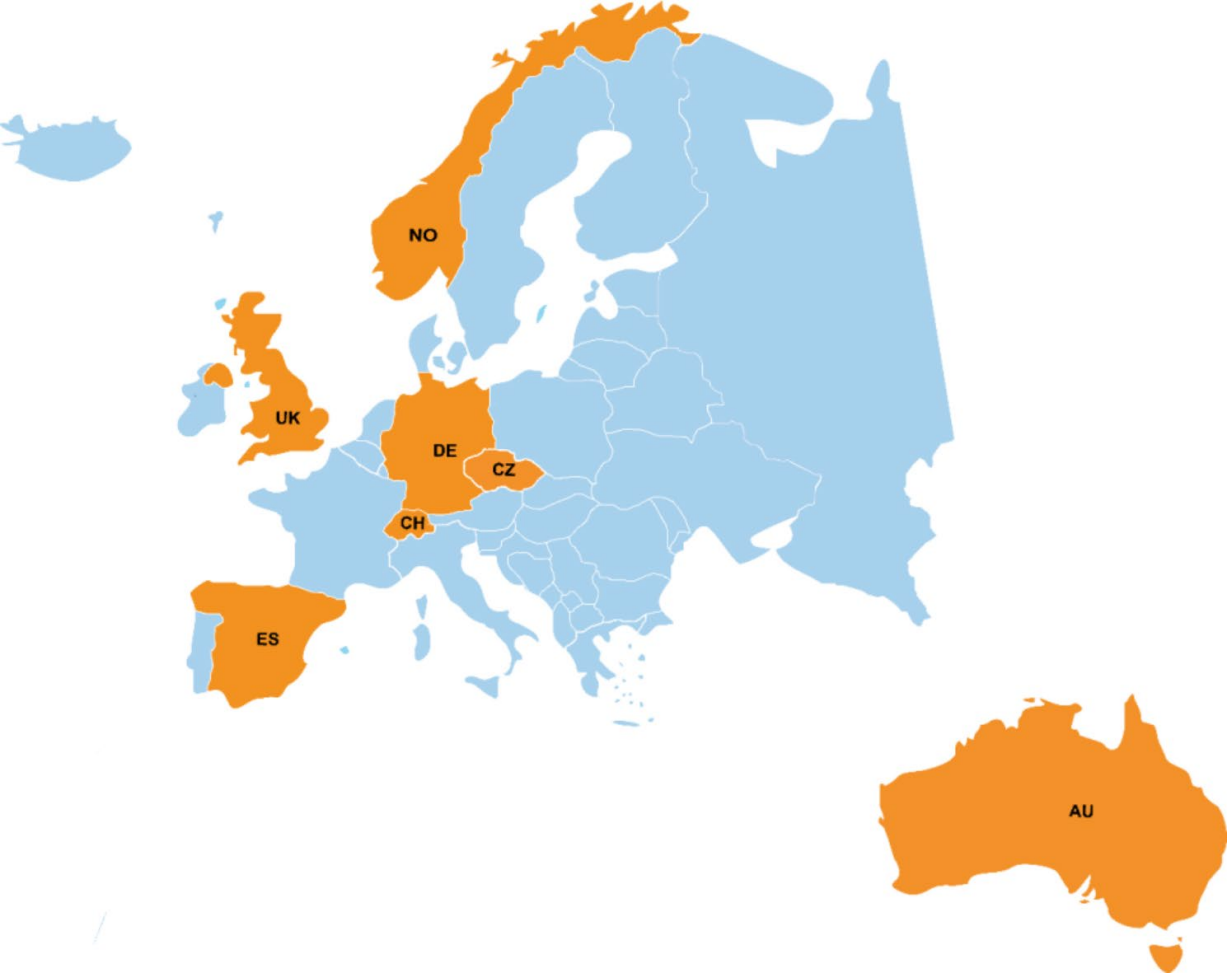
Countries selected for the analysis of child and adolescent mental health care strategies and models Abbreviations: AU – Australia; CH – Switzerland; CZ – Czechia; DE – Germany; ES – Spain; NO – Norway; UK – United Kingdom

For this scoping review, we tried to capture a broad variety of CAMH care strategies and models. We tried to achieve this by aiming for at least one country representing each European region (according to the UN Geoscheme for Europe [5]) and by representing countries with different health care systems and public health traditions. At the same time, we took into account transferability of the findings to the Austrian system (rationale: the original results were of particular interest for Austrian decision makers). This was done by restricting countries to those from the Global North and within them, selecting countries with the highest Human Development Index (HDI), 2020 report [6]. Considering this transferability, we excluded Asian countries due to the differences in health care systems and societal culture. Further, only countries who had documents available in English or German language and with a minimum population size of 5 million inhabitants were considered. This combination of criteria led to a set of six countries: Australia, Switzerland, Czechia, Spain, Norway, and the United Kingdom. In addition, Germany was included due to its proximity to Austria and high comparability of the health systems. The final set of selected countries is depicted in Fig. 1. Regarding the selection of documents within countries, the primary sources were national documents. If no national document was available, a limited number of regional documents were included. It was not the aim of our review to present a comprehensive country overview in quantitative terms (e.g., describing frequencies of certain characteristics of CAMH care models) but rather to capture the diversity and types of concepts.

### Document selection, data extraction and analysis of national strategies

From the literature search, we identified 128 national references. From these, we included national documents that describe a strategy or model of care specific for CAMH. In case no such specific document was available, we included a general mental health strategy or model that has a subchapter for children and adolescents. We excluded documents that were indication specific (e.g., depression, substance abuse, suicide). Further exclusion reasons were evaluation documents, surveys, situational analyses, or information campaigns.

We then prepared data extraction tables (available upon request) for each of the selected documents, deciding iteratively which information to extract. This process resulted in 14 topic areas for which we extracted information from the documents: information activities, prevention/promotion, detection, treatment, telemedicine, care pathways, transitional psychiatry, vulnerable patient groups, user participation, infrastructure, workforce development, implementation, digital tools for case management, and data acquisition/research. If available, we also extracted the documents’ key principles as well as conclusion sections. After all 14 topic areas were extracted from the documents, we reached our conclusion to the strategies by reviewing the findings and identifying patterns through qualitative content analyses.

For quality assessment of the included documents, we used an adapted version of the Appraisal of Guidelines for Research & Evaluation II (AGREE II) instrument [7]. This tool was used to assess six domains: scope & purpose, stakeholder involvement, rigor of development, clarity of presentation, applicability, and editorial independence. The process steps of quality assessment and data extraction were carried out by one researcher (RJ) and controlled by a second researcher (VH). In the process of adapting the preliminary categories for data extraction, a third researcher (IZ) was consulted for advice.

## Results

### Included national strategies

We included twelve relevant documents [8–19] from seven countries, published between 2013 and 2022, for our analysis of CAMH strategies and models. Table 1 gives an overview of included documents per selected countries.

**Table 1:** Overview of selected documents per country

Selected country	Included document(s)				
	Title	Publisher	Year	Language	Reference
**AU – Australia**	Head to Health Kids National Service Model	Australian Government Department of Health	2022	English	[8]
**AU – Australia**	The National Children’s Mental Health and Wellbeing Strategy	Australian Government	2021	English	[9]
**AU – Australia**	Child and Adolescent Mental Health Service Model of Care	Australian Capital Territory Government Health	2013	English	[10]
**CH – Switzerland**	Versorgungspfade in der psychiatrisch-psychotherapeutischen Versorgung von Kindern und Jugendlichen – SPD Basel	Swiss Confederation	2020	German	[11]
**CH – Switzerland**	Die Zukunft der Psychiatrie in der Schweiz	Swiss Confederation	2016	German	[12]
**CH – Switzerland**	Beabsichtigte Massnahmen zur psychischen Gesundheit in der Schweiz	Swiss Confederation	2016	German	[13]
**CH – Switzerland**	Psychische Gesundheit in der Schweiz, Bestandsaufnahme und Handlungsfelder	Swiss Confederation	2015	German	[14]
**CZ – Czechia**	National Mental Health Action Plan (NÁRODNÍ AKČNÍ PLÁN PRO DUŠEVNÍ ZDRAVÍ 2020–2030)	Ministry of Health of the Czech Republic	2020	Czech	[15]
**DE – Germany**	Further development of psychiatric-psychotherapeutic assistance and prevention of mental disorders in childhood and adolescence in Germany – development and coordination of recommendations for action („*Weiterentwicklung der psychiatrisch-psychotherapeutischen Hilfen und der Prävention seelischer Störungen im Kindes- und Jugendalter in Deutschland – Entwicklung und Abstimmung von Handlungsempfehlungen*“)	Aktion Psychisch Kranke e.V.	2021	German	[16]
**ES – Spain**	Roadmap: Recommendations for promoting mental health and emotional well-being in young people	Red PROEM (PROmotion of Mental Health and Emotional Well-being in the Young)	2018	English	[17]
**NO – Norway**	National guideline for health promotion and preventive work in the child and youth health centres and school health service, 0–20 years	Norwegian Directorate of Health	2020	English	[18]
**UK – United Kingdom**	Future in mind: promoting, protecting and improving our children and young people’s mental health and wellbeing	National Health Service England, Department of Health	2015	English	[19]

Abbreviations: NR – Not reported; SPD – Schulpsychologischer Dienst (eng.: School psychology service)

### Key principles

All included documents stated key principles on which CAMH services should be based. While these varied broadly across countries, most often mentioned key principles were to increase the focus on prevention, early detection/intervention (including efforts to improve mental health literacy), improved coordination and that CAMH services should be evidence-based including the need for more mental health services research. Additional key principles were to strengthen participation, to take special treatment needs into account, and to establish low-threshold services that are culturally sensitive and needs-based.

The general style of the documents differed in some regards. While some documents were rather strategic (Czechia, Germany, Spain), others were describing concrete models (Australia, Norway, United Kingdom) or a combination of both (Switzerland). Some documents explicitly provided an evidence base for each recommendation (e.g., Czechia, Norway), while others gave more jurisdictional context (Switzerland, Germany). One document in particular followed a thorough participatory approach, with the foreword of the document directed at children and adolescents in easy-to-understand language (United Kingdom).

### Topic areas in included documents

The majority, but not all of the 14 topic areas were described in each of the included documents. The document from Czechia addressed the fewest number of topic areas, while Australia and Spain were the only countries addressing all topic areas. Table 2 gives an overview which topic areas were described in the included documents.

**Table 2:** Topic areas in selected documents

Topic areas of model/strategy	Availability of information in selected document(s)						
	AU	CH	CZ	DE	ES	NO	UK
**Information, awareness raising activities**	✓	✓	✓	✓	✓	✓	✓
**Prevention, mental health promotion**	✓	✓	✓	✓	✓	✓	✓
**Detection, screening**	✓	✓	✓	NR	✓	✓	✓
**Treatment**	✓	✓	✓	✓	✓	✓	✓
**Digitalisation: Tools for detection, intervention, telehealth**	✓	NR	NR	✓	✓	✓	✓
**Care pathways, integrated care, health in all policies**	✓	✓	NR	✓	✓	✓	✓
**Transitional psychiatry**	✓	✓	NR	✓	✓	✓	✓
**Vulnerable patient groups**	✓	✓	NR	✓	✓	✓	✓
**Involvement, user participation**	✓	✓	NR	✓	✓	✓	✓
**Infrastructure, resources**	✓	✓	✓	✓	✓	✓	✓
**Professions, workforce development**	✓	✓	✓	✓	✓	✓	✓
**Implementation strategy, process**	✓	✓	✓	✓	✓	✓	✓
**Digitalisation: Tools for case management and documentation**	✓	✓	NR	✓	✓	NR	NR
**Data acquisition, research**	✓	✓	NR	✓	✓	✓	✓
**Overall**	**14**	**13**	**7**	**13**	**14**	**13**	**13**

Abbreviations: AU – Australia, CH – Switzerland, CZ – Czechia, DE – Germany, ES – Spain, NO – Norway, NR – not reported, UK – United Kingdom

### Information activities, prevention/promotion, detection

All countries recommend information and awareness raising activities to increase health literacy and reduce stigma. Several distribution channels (traditional and social media), targeting children, adolescents, carers, and less often professionals, are recommended. For strengthening mental health promotion and illness prevention, the focus is on increasing early help-seeking, and improving interpersonal relationships (e.g., reducing violence and improving respectfulness). One key target group of these activities are parents with a mental illness, as there is rising awareness of the negative impact parental mental illness can have on children. One recommendation in this area is to introduce specialised programmes aimed at developing parenting skills, especially for families under psychosocial stress, in order to reduce the risk of traumatisation of children. Another recommendation is to increase care for these affected families, to counteract the existing disadvantages. The key setting of promotion and prevention activities is within schools. Similarly, the recommended core setting for screening and early detection in the documents is the school environment, involving different professionals (e.g., school health nurses).

### Treatment, telemedicine, care pathways, transitional psychiatry

Regarding treatment, the necessity to broaden the range of settings is highlighted, ranging from home-treatment, inpatient-equivalent treatment and other outreach approaches, to inpatient treatment. To allow family-focused care, alternatives to hospital care are described as important. As for medication, the need to improve safety by installing measures to reduce the wide off-label use is emphasised. Suggestions on the mode of care delivery include telehealth and face-to-face approaches in single- and/or group settings. The increased use of digital applications is mainly recommended for detection, self-care, and better system navigation. Australia has defined the expansion of telehealth tools as a priority activity, however, with funding extensive evaluation research alongside.

Suggestions on care pathways and integrated care play a prominent role aiming at integrating services across different sectors, such as health and education. A key suggestion is to establish a single point of access and to provide a coordinator for each patient/family. The pathways to access hospital care should be clearly defined and linked to quality indicators monitoring waiting times or “no shows”. The care pathways are recommended to be in line with the different developmental stages throughout child and adolescence, e.g., which service networks and interventions are required at each stage of development. For better management of transitional phases (e.g., when entering schools, or from adolescent to adult psychiatry), a shared recommendation is to move away from age thresholds to needs-based transitions depending on developmental stages, including extending ages for transition into adult mental health care up to 25 years.

### Vulnerable patient groups, user participation, infrastructure

A broad number of especially vulnerable groups, at greater risk for developing a mental illness and often requiring complex care arrangements, have been identified across all documents. These include culturally and linguistically diverse groups. Measures to better and routinely detect circumstances that make a child vulnerable and installing a lead professional for case management (shifting the responsibility for coordination from the family to the professionals) are recommended. Further, an additional re-distributing of resources to better care for these vulnerable children and adolescents are suggested. These resources are recommended to cover interpreter services, additional time, personnel, and material resources, developing social and emotional wellbeing services for diverse populations, and appropriate referrals to specialist mental health services. Another topic described across most documents is strengthened user participation. The aim is to provide more tailored care and better navigation in care pathways, by involving users in both individual care planning but also in the systemic design processes of the services.

Regarding infrastructure and resources, the countries recommend infrastructure to be accessible and culturally safe. For financing, an increase in budget is required for interprofessional collaboration, psychotherapy and psychoeducational programmes, and for policies aimed at preventing inequality in youth. Examples are ring-fenced budgets jointly provided by the health, social and educational ministry. Additionally, new reimbursement mechanisms for providers are suggested to fund a combination of outpatient services for children with complex needs.

### Workforce development, implementation, digital tools, data acquisition/research

The workforce qualification and professional development is another topic area described across most documents. Most countries suggest broadening the traditional health workforce with additional workers (e.g., allied health specialists, family therapists, diverse and LGBTIQ + health workers, peer support workers). A specific highlight is put on developing the competences of school teaching staff (led by mental health specialists), as well as implementing a designated wellbeing staff member in all schools. For the implementation strategy and process, some countries created inter-ministerial/cross-sectional committees, with individual responsibilities from managers of health centers, school health services and municipalities. To guide implementation, it is recommended to identify what is working in some regions and rolling it out to the whole country, with phases of implementation described as an establishment phase, an embedding phase, and a full operational phase.

Almost all countries recommend increasing data acquisition and research with the aim of monitoring changes in mental disorder prevalence and effects of interventions. A broad range of specific research topics and study designs are listed, including (cross-sectoral) mental health service research allowing benchmarking of services (e.g., waiting times and user satisfaction). Several countries are in the process of implementing national mental health services datasets for data acquisition and sharing across relevant sectors. Most countries further suggest increasing the availability of digital tools for case management (e.g., tools to facilitate clinical decision making and cross-sector video conferences) and documentation (e.g., continued development of trans-organisational electronic patient record systems).

### Establishing a child and adolescent mental health strategy

**Fig. 2:**
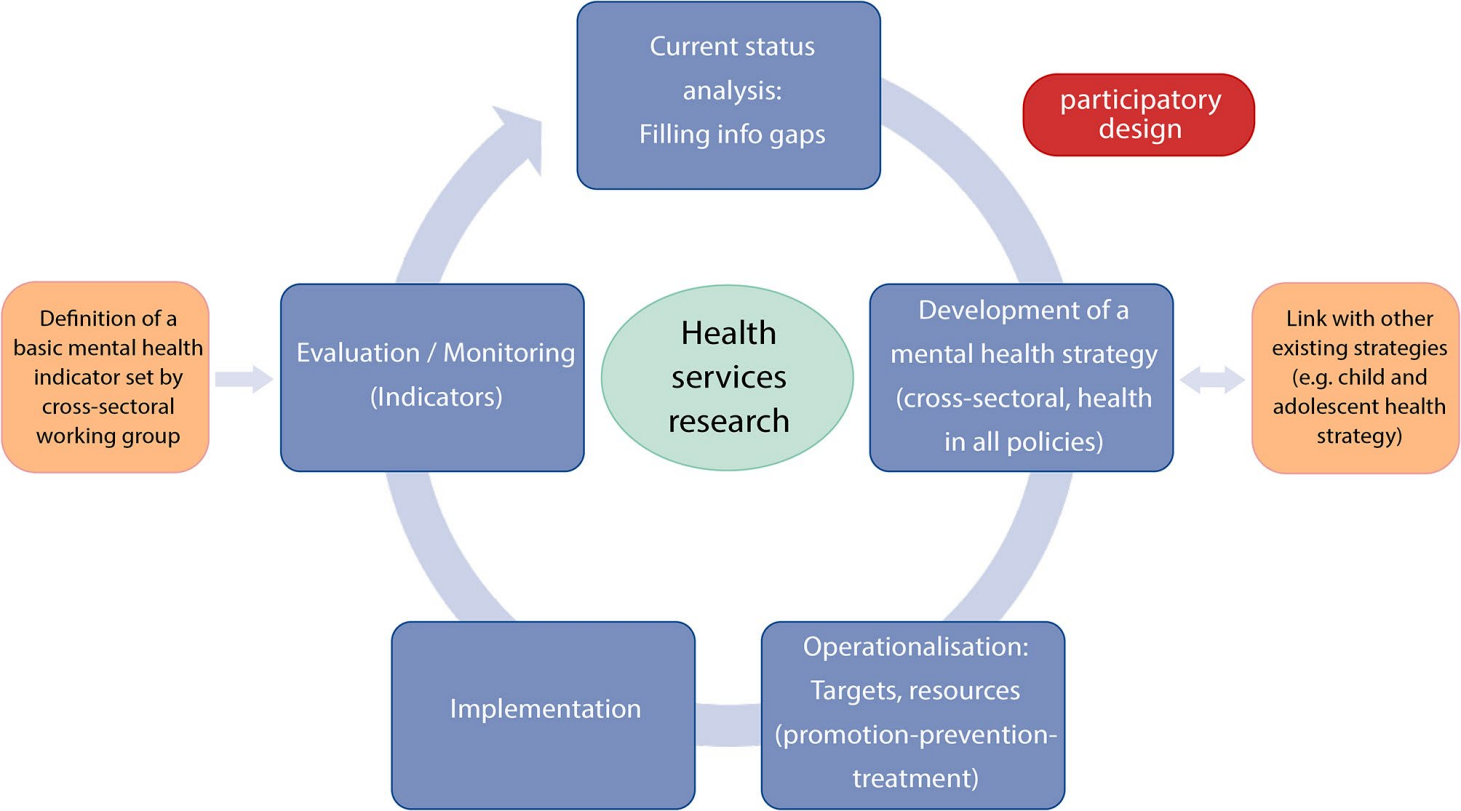
Guiding principles for a stand-alone child and adolescent mental health strategy

Drawing inspiration from the recommendations in international CAMH strategies and models, the following Fig. 2 may serve as a starting point for health policy discussions on establishing and further adapting a stand-alone CAMH strategy. In this guiding principle, the importance of a participatory design approach is emphasised. Further, health services research plays a central role for the depicted components (current status analysis, development of a mental health strategy, operationalisation, implementation, and evaluation).

### Quality assessment

The assessment of the overall quality of the documents ranged from 62 to 95%. Reasons for lower ratings were, among others: the views and preferences of the target population were not always included, the target users of the documents were not always clearly defined, and no application of systematic methods to search for evidence, and the recommendations were not always explicitly linked to supporting evidence (see Table 3).

**Table 3:** Quality assessment of the included documents

Quality Assessment Check	AU, 2022 [8]	AU, 2021 [9]	AU, 2013 [10]	CH, 2020 [11]	CH, 2016 [12]	CH, 2016 [13]	CH, 2015 [14]	CZ, 2020 [15]	DE, 2021 [16]	ES, 2018 [17]	NO, 2020 [18]	UK, 2015 [19]
**Domain 1: Scope and Purpose**												
1.The overall objective(s) of the guideline [document] is (are) specifically described.	7	7	7	7	7	4	5	7	7	7	7	7
2.The health question(s) covered by the guideline is (are) specifically described.	NA	NA	NA	NA	NA	NA	NA	NA	NA	NA	NA	NA
3.The population (patients, public, etc.) to whom the guideline [document] is meant to apply is specifically described.	7	6	7	6	5	5	5	6	6	6	7	6
**Domain 2: Stakeholder Involvement**												
4.The guideline [document] development group includes individuals from all the relevant professional groups.	7	7	6	7	7	7	7	6	7	7	7	7
5.The views and preferences of the target population (patients, public, etc.) have been sought.	7	7	7	5	3	3	3	3	6	7	7	6
6.The target users of the guideline [document] are clearly defined.	4	4	4	4	5	4	4	4	7	7	7	7
**Domain 3: Rigour of Development**												
7.Systematic methods were used to search for evidence.	1	1	1	1	1	1	1	1	1	1	3	1
8.The criteria for selecting the evidence are clearly described.	NA	NA	NA	NA	NA	NA	NA	NA	NA	NA	NA	NA
9.The strengths and limitations of the body of evidence are clearly described.	NA	NA	NA	NA	NA	NA	NA	NA	NA	NA	NA	NA
10.The methods for formulating the recommendations are clearly described.	NA	NA	NA	NA	NA	NA	NA	NA	NA	NA	NA	NA
11.The health benefits, side effects, and risks have been considered in formulating the recommendations.	NA	NA	NA	NA	NA	NA	NA	NA	NA	NA	NA	NA
12.There is an explicit link between the recommendations and the supporting evidence.	4	7	4	3	7	3	5	6	3	4	7	5
13.The guideline has been externally reviewed by experts prior to its publication.	NA	NA	NA	NA	NA	NA	NA	NA	NA	NA	NA	NA
14.A procedure for updating the guideline is provided.	NA	NA	NA	NA	NA	NA	NA	NA	NA	NA	NA	NA
**Domain 4: Clarity of Presentation**												
15.The recommendations are specific and unambiguous.	NA	NA	NA	NA	NA	NA	NA	NA	NA	NA	NA	NA
16.The different options for management of the condition or health issue are clearly presented.	NA	NA	NA	NA	NA	NA	NA	NA	NA	NA	NA	NA
17.Key recommendations are easily identifiable.	6	7	6	5	7	5	6	6	7	7	7	7
**Domain 5: Applicability**												
18.The guideline [document] describes facilitators and barriers to its application.	5	6	4	5	7	5	6	6	6	5	7	7
19.The guideline provides advice and/or tools on how the recommendations can be put into practice.	NA	NA	NA	NA	NA	NA	NA	NA	NA	NA	NA	NA
20.The potential resource implications of applying the recommendations have been considered.	5	6	4	6	7	6	6	7	7	6	7	7
21.The guideline [document] presents monitoring and/or auditing criteria.	7	7	6	5	7	5	5	6	7	6	7	6
**Domain 6: Editorial Independence**												
22.The views of the funding body have not influenced the content of the guideline.	NA	NA	NA	NA	NA	NA	NA	NA	NA	NA	NA	NA
23.Competing interests of guideline development group members have been recorded and addressed.	NA	NA	NA	NA	NA	NA	NA	NA	NA	NA	NA	NA
**Overall quality of this guideline/document** (1 – lowest possible quality, 7 – highest possible quality)	**60 of 77 (78%)**	**65 of 77 (84%)**	**56 of 77 (73%)**	**54 of 77 (70%)**	**63 of 77 (82%)**	**48 of 77 (62%)**	**53 of 77 (69%)**	**58 of 77 (75%)**	**64 of 77 (83%)**	**63 of 77 (82%)**	**73 of 77 (95%)**	**66 of 77 (86%)**

Rating Scale: 1 – Strongly Disagree; 7 – Strongly Agree

Abbreviations: AU – Australia; CH – Switzerland; CZ – Czechia; DE – Germany; ES – Spain; NA – not applicable;

NO – Norway; NR – not reported; UK – United Kingdom

## Discussion

### Main findings

We found that all countries included in this report advocate a public mental health approach that is characterised by encouraging mental health promotion and prevention. For this aim, the educational sector is essential and should be supported with additional staff and mental health training for educators. In this cross-sectoral approach, in terms of professionals involved and their responsibilities, CAMH is shifting from a psychiatry-focused (more medical-oriented) model to multi-professional teams with shared responsibility and equal contributions from different professional groups.

### Interpretation

Mental health policies are a logical first step in recognising need for care and prevention, and subsequent programme development. Mental health policies are guides for actions, such as programme development, financing, and access to care [20]. In this context, all the countries selected for our analysis have a general mental health strategy and, in the majority, additionally a CAMH strategy and a suicide prevention strategy exists. For adopting CAMH services, the documents suggest that prevention and care should be based on the needs of children and their families, rather than on the existing care structures. This needs-based approach increases the relevance of user participation and involvement, focusing on special support for vulnerable groups, and through creating structures within CAMH services that promote coordination of services according to individual needs (e.g., single points of access, coordinators for improved system navigation).

Based on the recommendations from the national documents, a CAMH strategy with a common vision and shared goals, integrating mental health promotion, illness prevention and mental health care across relevant sectors (health, social care, education, and criminal justice) is advisable. In particular, strengthening the outpatient area (e.g., home-treatment services) to overcome hospital bed and psychiatrist shortage, as well as informal care offers in flexible settings with improved care pathways is essential. This extension would also be more in line with the optimal mix of mental health services as recommended by the World Health Organization. A CAMH strategy needs to be linked with other existing strategies (e.g. child and adolescent health strategy). An aspect that was barely mentioned in the included documents is the role of perinatal and infant mental health. However, when designing a CAMH service, insights from perinatal and infant mental health care models and pathways can inform care models in the older ages [21]. The need for increased focus on vulnerable patient groups becomes evident when considering recent research findings, such as the multinational EU-GEI study. In this study, social vulnerability due to migration (and post-migration experiences) was associated with double the odds of psychosis in first-generation migrants [22]. An ideal CAMH service addresses the issues of culturally and linguistically diverse groups, by putting increased focus on these vulnerable patient groups. Interpretation services are important, but additional measures addressing this patient group are also required. These include promoting respect and reducing stigma towards social minority groups, and increasing adolescents’ empathy and tolerance towards what is different and diverse. For this purpose, measures are called for to reduce inequalities, such as redistribution of existing therapeutic resources towards psychosocial and/or economically challenged families.

Furthermore, the recommendations from the national documents are in accordance with the results from the ROAMER project (ROAdmap for MEntal health Research in Europe). The defined priorities regarding public mental health from an expert consensus fall in three overarching goals: to identify causes, risk and protective factors for mental health across the lifespan; to advance the implementation of effective public mental health interventions; and to reduce disparities in mental health [23].

A particular role in further improving CAMH is within the educational sector. While a comprehensive integration of mental health and mental disorder topics in the teaching content is recommended, additional school staff (e.g., well-being staff) and further development of the mental health competences for school teaching staff, guided by mental health specialists, is a key recommendation in the included documents. The school setting is essential for information activities and reduction of stigma, and to further achieve the goal of an open-minded and strengths-based (rather than deficit-oriented) approach to mental health, focusing on child’s functioning (rather than on the diagnosis). School-based interventions for CAMH can yield moderate to strong effects for a range of emotional and behavioural problems when implemented with evidence-based cognitive behavioural interventions [24]. Regarding adolescents at risk of suicide, mental health literacy programmes in the school setting (such as the Teen Mental Health First Aid) aim to increase recognition and support towards affected peers [25].

The recommendations in the documents are not always supported with evidence and implementation requirements. For example, it is unclear if there are any drawbacks to moving away from age as a transition criterion and what the training requirements of implementing the suggested approach would be. Current research projects, for example the preparation of a clinical research facility for transitional psychiatry in Austria [26], could provide the basis for implementation studies or outcome studies of such an adapted model of care.

Furthermore, the countries recommend additional health service research, implementing a national mental health dataset for coordinated data acquisition and increasing the availability of digital tools for management and documentation. In this context, indicators for planning CAMH services and monitoring CAMH can be utilised to create a better way of dealing with current shortcomings in care and prevention [27, 28]. A report from the United Nations Children’s Fund (UNICEF) highlights that routine monitoring of mental health and mental healthcare is seriously lacking, especially for CAMH [29]. As part of our own research, a collection of internationally identified indicators covering the broad spectrum of mental health (such as risk factors, prevention and promotion factors, life satisfaction, supply and utilization of services, quality of care, and sociodemographic indicators) is publicly available [30]. It may serve as a starting point for defining core-indicator sets.

### Limitations

For identifying data in our study, we used a targeted hand search (instead of a systematic literature search). We expected that CAMH care models and strategies are usually not published in scientific journals, but rather as grey literature on relevant websites (e.g., ministerial, or public health). As other authors might have identified a different set of documents for analysis, our research findings are not fully reproducible. In addition, we were only able to consider a selection of countries based on the HDI as an indicator. As the HDI combines three dimensions we considered it a better fit for our study purpose compared to other indicators, such as gross domestic product (GDP). However, other indicators could have also been used for country selection.

Further, excluding Asian countries is a limitation, as there are Asian countries with a higher HDI rank than those included in our study, with healthcare systems comparable to western systems (such as Japan or Singapore). Due to the selection strategy, and to the literature search being limited to June 2022, there is a possibility that other (or novel) CAMH policies or models exist that are not included in our study. Furthermore, we did not collect data on the extent to which the strategies have already been implemented in the selected countries.

We excluded documents addressing specific indications (e.g., medical guidelines), general health (instead of specific to mental health), or all ages (instead of specific to children and young people). These excluded documents might contain some supplementary information.

Furthermore, when extracting data from the documents, the information on the identified topic areas could not always be clearly assigned to the identified categories due to overlaps. Many aspects of mental health are intertwined, and although we have tried to match them carefully with the topic areas, there may have been alternative ways of assignment.

## Conclusion

Based on our findings, we strongly advocate the use of cross-sectoral and participatory approaches for CAMH care structures. The involved sectors (health, education, social and youth justice) should integrate mental health promotion, prevention and care in a shared vision. For this purpose, health service research should accompany the strategic developments, aiming to create better ways of dealing with shortcomings in care in general, and for vulnerable patient groups specifically.

## Data Availability

The datasets generated and/or analysed during the current study can be made available from the corresponding author upon request.

## References

[1] Polanczyk GV, Salum GA, Sugaya LS, Caye A, Rohde LA (2015). Annual Research Review: a meta-analysis of the worldwide prevalence of mental disorders in children and adolescents. J Child Psychol Psychiatry.

[2] Ravens-Sieberer U, Kaman A, Otto C, Adedeji A, Napp A-K, Becker M (2021). Seelische Gesundheit und psychische Belastungen Von Kindern Und Jugendlichen in Der Ersten Welle Der COVID-19-Pandemie – Ergebnisse Der COPSY-Studie. Bundesgesundheitsblatt - Gesundheitsforschung - Gesundheitsschutz.

[3] World Health Organization. WHO MiNDbank: More Inclusiveness Needed in Disability and Development - A database of resources covering mental health, substance abuse, disability, general health, human rights and development. 2022 [cited 18.10.2022]. Available from: https://extranet.who.int/mindbank/collection/country.

[4] European Commission. Youthwiki: Europe encyclopedia of National Youth Policies. 2022 [cited 27.09.2022]. Available from: https://national-policies.eacea.ec.europa.eu/youthwiki.

[5] United Nations Statistics Division. Methodology - Standard country or area codes for statistical use. 2022 [cited 18.10.2022]. Available from: https://unstats.un.org/unsd/methodology/m49/.

[6] United Nations Human Development Reports. Human Development Index (HDI). 2020 [cited 01.09.2022]. Available from: https://hdr.undp.org/data-center/human-development-index#/indicies/HDI.

[7] Brouwers MC, Kho ME, Browman GP, Burgers JS, Cluzeau F, Feder G (2010). AGREE II: advancing guideline development, reporting and evaluation in health care. CMAJ.

[8] Australian Government Department of Health and Aged Care. Head to Health Kids National Service Model. 2022 [cited 27.09.2022]. Available from: https://www.health.gov.au/sites/default/files/documents/2022/08/head-to-health-kids-national-service-model_0.pdf.

[9] Australian Government. The National Children’s Mental Health and Wellbeing Strategy. 2021 [cited 27.09.2022]. Available from: https://www.mentalhealthcommission.gov.au/getmedia/5b7112be-6402-4b23-919d-8fb9b6027506/National-Children%E2%80%99s-Mental-Health-and-Wellbeing-Strategy-%E2%80%93-Report.

[10] ACT Government Health, Child, and Adolescent Mental Health Service Model of Care. 2013 [cited 27.09.2022]. Available from: https://health.act.gov.au/sites/default/files/2018-09/Child%20and%20Adolescent%20Mental%20Health%20Service%20Model%20of%20Care%20%28May%202013%29.pdf.

[11] Schweizerische Eidgenossenschaft. Versorgungspfade in der psychiatrisch-psychotherapeutischen Versorgung von Kindern und Jugendlichen – SPD Basel. 2020 [cited 27.09.2022]. Available from: https://www.bag.admin.ch/dam/bag/de/dokumente/berufe-gesundheitswesen/Interprofessionalitaet/Forschungsberichte1/studie-m19-versorgungspfade-spd-basel-schlussbericht.pdf.download.pdf/Studie%20M19_Versorgungspfade%20in%20der%20psychiatrisch-psychotherapeutischen%20Versorgung%20von%20Kindern%20und%20Jugendlichen_SPD%20Basel_Schlussbericht.pdf.

[12] Schweizerische Eidgenossenschaft. Die Zukunft der Psychiatrie in der Schweiz. 2016 [cited 27.09.2022]. Available from: https://sbap.ch/wp-content/uploads/2017/06/Bericht_Zukunft_Psychiatrie_DE.pdf.

[13] Schweizerische Eidgenossenschaft. Beabsichtigte Massnahmen zur psychischen Gesundheit in der Schweiz. 2016 [cited 27.09.2022]. Available from: https://www.bag.admin.ch/dam/bag/de/dokumente/cc/bundesratsberichte/2016/psychische-gesundheit.pdf.download.pdf/psychische-gesundheit.pdf.

[14] Schweizerische Eidgenossenschaft. Psychische Gesundheit in der Schweiz, Bestandsaufnahme und Handlungsfelder. 2015 [cited 27.09.2022]. Available from: https://gesundheitsfoerderung.ch/assets/public/documents/de/5-grundlagen/publikationen/psychische-gesundheit/Bericht_Psychische_Gesundheit_in_der_Schweiz_-_Bestandsaufnahme_und_Handlungsfelder.pdf.

[15] Ministry of Health of the Czech Republic (Ministerstvo zdravotnictví České republiky). National Mental Health Action Plan (NAPDZ - NÁRODNÍ AKČNÍ PLÁN PRO DUŠEVNÍ ZDRAVÍ 2020–2030). 2020 [cited 27.09.2022]. Available from: https://www.mzcr.cz/wp-content/uploads/2020/01/N%C3%A1rodn%C3%AD-ak%C4%8Dn%C3%AD-pl%C3%A1n-pro-du%C5%A1evn%C3%AD-zdrav%C3%AD-2020-2030.pdf.

[16] Aktion Psychisch Kranke e.V. Weiterentwicklung der psychiatrisch-psychotherapeutischen Hilfen und der Prävention seelischer Störungen im Kindes und Jugendalter in Deutschland – Entwicklung und Abstimmung von Handlungsempfehlungen. 2021 [cited 27.09.2022]. Available from: https://www.apk-ev.de/fileadmin/downloads/Materialien_KiJu/Abschlussbericht_APK-Projekt_KiJu-WE_.pdf.

[17] Red PROEM. Roadmap: Recommendations for promoting mental health and emotional well-being in young people. 2018 [cited 27.09.2022]. Available from: https://redproem.es/wp-content/uploads/2018/06/Informe_encuentro_Red_PROEM_EN.pdf.

[18] Norwegian Directorate of Health. National guideline for health promotion and preventive work in the child and youth health centres and school health service, 0–20 years. 2020 [cited 27.09.2022]. Available from: https://www.helsedirektoratet.no/retningslinjer/helsestasjons-og-skolehelsetjenesten/dokumenter-helsestasjons-og-skolehelsetjenesten/National%20guideline%20for%20health%20promotion%20and%20preventive%20work%20in%20the%20child%20.pdf.

[19] National Health Service England D. o. H. Future in mind: promoting, protecting and improving our children and young people’s mental health and wellbeing. 2015 [cited 27.09.2022]. Available from: https://assets.publishing.service.gov.uk/government/uploads/system/uploads/attachment_data/file/414024/Childrens_Mental_Health.pdf.

[20] Shatkin JP, Balloge N, Belfer ML (2008). Child and adolescent mental health policy worldwide: an update. Int Psychiatry.

[21] Reinsperger I, Paul J. Perinatal and infant mental health care models and pathways. A scoping review. AIHTA Project Report No.: 148; 2022. Vienna: HTA Austria - Austrian Institute for Health Technology Assessment GmbH.: 2022 [cited 30.08.2023]. Available from: https://eprints.aihta.at/1420/1/HTA-Projektbericht_Nr.148.pdf.

[22] Tarricone I, D’Andrea G, Jongsma HE, Tosato S, Gayer-Anderson C, Stilo SA (2022). Migration history and risk of psychosis: results from the multinational EU-GEI study. Psychol Med.

[23] Forsman AK, Wahlbeck K, Aarø LE, Alonso J, Barry MM, Brunn M (2015). Research priorities for public mental health in Europe: recommendations of the ROAMER project. Eur J Pub Health.

[24] Paulus FW, Ohmann S, Popow C (2016). Practitioner review: School-based interventions in child mental health. J Child Psychol Psychiatry.

[25] Hart LM, Cropper P, Morgan AJ, Kelly CM, Jorm AF (2019).

[26] Ludwig Boltzmann Gesellschaft - Open Innovation in Science Center. Klinisches Forschungszentrum für Transitionspsychiatrie. 2023 [cited 01.09.2023]. Available from: https://ois.lbg.ac.at/projekte/klinisches-forschungszentrum-fuer-transitionspsychiatrie/.

[27] Peitz D, Kersjes C, Thom J, Hoelling H, Mauz E. Indicators for Public Mental Health: a scoping review. Front Public Health. 2021;9. 10.3389/fpubh.2021.714497 PMC8502920 34646802

[28] UNICEF. Adolescent mental health indicators. 2021 [cited 12.06.2022]. Available from: https://data.unicef.org/resources/adolescent-health-indicators/.

[29] UNICEF. The State of the World’s Children. 2021 - On My Mind - Promoting, protecting and caring for children’s mental health. 2021 [cited 18.10.2022]. Available from: https://www.unicef.org/media/114636/file/SOWC-2021-full-report-English.pdf.

[30] Jeindl R, Hofer V. Child and adolescent mental health care models. A scoping review. AIHTA Project Report No.: 149. Vienna: HTA Austria - Austrian Institute for Health Technology Assessment GmbH (2022). 2022 [cited 12.02.2023]. Available from: https://eprints.aihta.at/1418/.

